# Clinics in Bellevue

**Published:** 1871-10

**Authors:** C. H. Richmond

**Affiliations:** New York


### Correspondence.

### Clinics in Bellevue.

New York, October 17, 1871.

*To the Editor of the Buffalo Medical and Surgical Journal.*

My Dear Doctor:—Thinking you might like to know a little of what is taking place in the way of medical teaching here, I will give you a concise idea of some of the cases presented at the clinics of the Professors in the Bellevue Medical College. I will first state, however, that Prof. White, of the Buffalo College, attended one of the clinics of Dr. Flint a few days ago, and was received with most hearty applause by those who enjoyed the advantages of his instruction here last winter.

In a Surgical point of view, this College affords some advantages in having a large number of teachers; and during a whole course of lectures, no doubt cases will be observed illustrating nearly all diseases with many of the operations. But there are some disadvantages to the student which do not obtain in colleges less largely attended. It is impossible for all to see the varied steps of an operation, and students are not permitted to pass through the wards of the hospitals, so that in reality a much less number of cases is seen, with their variations and complications, than in some of our inland institutions.

Dr. Hamilton presented, some two or three weeks ago, a case of actual scirrhus of the breast, which he treated by Electrolysis. The patient had suffered from much pain, and there was enlargement of some of the axillary glands. At the present time the tumor is reduced in size, and softer, the axillary glands are diminishing in size and hardness, and the patient has not suffered from a particle of pain since the operation. The doctors inference from this and a few other cases is, that while electricity by means of needles will not eliminate the essential nature of cancer or affect its tendency to become localized, it has the power of dispelling the tumor consequent upon its influence, and if no other result be obtained than the relief of pain, and the consequent nutrition of the body it is a sufficient reason for employing it. He did not say he would substitute it for the knife in cases falling under the rules for operative procedure.

Dr. Stephen Smith had a case of dislocation of the shoulder backward, a rare form of injury, caused by a direct blow upon the front of the upper end of the humerus. The injury had been received several days previous, and some difficulty attended the reduction. Manipulation failed to reduce the bone, owing, the doctor thought, to rupture of the subscapularis tendon, thereby losing the benefit of its assistance. The arm was then drawn somewhat obliquely across the patients chest, and with strong traction made by an assistant, the doctor at the same time pushing against the head of the bone with some manipulations, the reduction was finally accomplished. This was an interesting case to me, as it was the second case I ever saw, the first being a recent case by Dr. Hamilton in the Buffalo Hospital of the Sisters of Charity I believe in 1859. Reduction in that case was not attended with difficulty.

Dr. Gouley, (not of the Bellevue College) at the Bellevue Hospital, ligated the primitive (?) illiac on the 12th inst., for "true diffused" aneurism of the external illiac, assisted by Drs. Mott, Markoe, and Crane. Dr. Van Buren and others were also present,

He performed Crampton's operation, by cutting from near the termination of the eleventh rib to the neighborhood of the anterior superior spinous process of the illium to the peritoneum, passing under which until he found the artery and secured it. The doctor dared not pass far downward for fear of injury to the tumor which was large, and he was in doubt as to which artery he had reached viz.: the external or primitive illiac. Dr. Mott was fully of the opinion that it was the primitive from its Surgical relations, and a recent aneurismal tumor would not be capable of pushing the artery from its bed. The patient at the present time is doing well.

Dr. Sayer has showed us the practical application of his apparatus for extending the limb in diseased joints, still enabling the patient to go about. His vertebrated probe seems indispensible in the diagnosis of certain cases of diseased bone, the seat of which can only be reached by crooked sinuses. His views attracted a good deal of attention in Europe while there during the past summer, and were received with enthusiasm.

In the clinics for diseases of women, Dr. Isaac E. Taylor has presented a case of a colored girl 21 years old, which he thinks is a clear case of spontaneous inversion. The length of the cavity has been found by measurement to have been at one time six inches with lengthening and enlargement of the cervix. It was attended by procidentia dilating and pressing through the hymen, which is intact, and vulva. It has spontaneously undergone a nearly complete return to its normal length, though the cervix is still only just within the hymen, proving, the doctor thinks, that the virgin uterus may undergo spontaneous inversion and repair. It is probable, however, that most teachers of gynæcology would not admit that this case was more than one of the lining membrane from inertia with descent of the whole organ. Dr. Taylor has also presented some interesting cases of lupus of the vulva.

Dr. Flint presents his cases with his usual clearness, clearing up the diagnosis of doubtful cases to the satisfaction of his listeners.

There is much more that I could write about in a clinical way but time and space forbid; nor have I thought best to comment on the procedures, etc., which I have related.

Very respectfully,

C. H. RICHMOND.

